# Risk factors for maladaptive grief and posttraumatic stress in children and adolescents following loss: a systematic review

**DOI:** 10.1186/s13034-026-01076-7

**Published:** 2026-03-29

**Authors:** Philipp Jann, Neele Winkler, Harald Karutz, Veronika Hulde, Tobias Hecker

**Affiliations:** 1 Department of Clinical Psychology and Violence Research, Bielefeld University, Universitätsstraße 25, Postbox 100131, 33615 Bielefeld, Germany; 2 Institute for Emergency Psychology, Bielefeld, Germany; 3 Institute for Psychosocial Crisis Management, MSH Medical School Hamburg, Am Kaiserkai 1, 20457 Hamburg, Germany; 4 Institute for Interdisciplinary Research on Conflict and Violence, Bielefeld University, Universitätsstraße 25, 33615 Bielefeld, Germany

**Keywords:** Loss, Childhood, Adolescence, Prolonged grief disorder, Posttraumatic stress disorder, Risk factors, Systematic review

## Abstract

**Background:**

Traumatic losses in childhood and adolescence can evoke both maladaptive grief symptoms (MGS) and posttraumatic stress symptoms (PTSS). The aim of this study was to systematically review the existing literature to identify which risk factors are shared by both phenomenologies or differentiate between them.

**Methods:**

The systematic review was pre-registered in PROSPERO and conducted in accordance with PRISMA guidelines. A systematic literature search was performed in PubMed, Medline, PsycInfo, and Web of Science, identifying 18 studies based on 14 samples that met the inclusion criteria. A quality assessment was performed using the JBI critical appraisal checklist.

**Results:**

Encompassing a total of 5,034 individuals, the samples were generally gender-balanced (*M* = 52% female, *SD* = 14%) with a mean age of 12.88 years (*SD* = 2.19). The risk factors were summarised in six superordinate clusters. Intrapersonal reactions were very often significantly associated with both MGS and PTSS. In addition, other mental health problems were examined in most studies and were significantly associated with PTSS and MGS in all of these studies, indicating a very high relevance. We could draw no conclusion regarding demographic factors and the relevance of loss characteristics and circumstances. The quality of the included studies was good, but only three studies were longitudinal.

**Conclusions:**

The study emphasizes the relevance of intrapersonal factors and other comorbidity, with detailed discussion of their implications. Improving the specific identification of children and adolescents with high-risk profiles would particularly benefit areas with few resources for care. Due to the relevance of the topic, there is an urgent need for more studies investigating MGS and PTSS following loss in childhood and adolescence in the same sample.

**Supplementary Information:**

The online version contains supplementary material available at 10.1186/s13034-026-01076-7.

## Introduction

The death of relatives or friends is one of the most critical life events children and adolescents can experience. In particular, the death of a parent weighs especially heavily (Alvis et al. 2023; [38]). Such an experience of loss increases the likelihood of short-term and long-term mental and physical illness significantly (e.g., [13, 21, 24]) and even decreases life expectancy [68]. These effects appear to be even more severe if the loss is traumatic in nature [3, 67]. Such losses are often referred to as traumatic losses [36]. On the one hand, they can be objectively assessed by the circumstances of death, such as homicide, suicide, accidents, war, terror or natural disasters [11]. On the other hand, subjective evaluations of the loss, for example if it is perceived as particularly sudden, violent or unjust, can also be considered potentially traumatic losses [6].

A traumatic loss can evoke maladaptive grief symptoms (MGS) or posttraumatic stress symptoms (PTSS), which may persist and develop into Prolonged Grief Disorder (PGD) or Posttraumatic Stress Disorder (PTSD). While PTSD is primarily characterised by intrusion, avoidance and persistent hyperarousal due to the stressful event, PGD is primarily characterised by intense yearning for the deceased person and preoccupation with thoughts or memories of the deceased person (DSM-5-TR; [5]).

Scientific debates concerning the differentiation between adaptive and maladaptive grief have been shaped by various terminological proposals in recent years (e.g., “Traumatic Grief”, [64], “Complicated Grief”, Shear et al., 2011; “Persistent Complex Bereavement Disorder”, APA, 2013). However, these approaches have consistently converged in their core criteria to differentiate normal from maladaptive grief: (a) the loss of a close relative; (b) yearning; and (c) cognitive, emotional, and behavioral symptoms, such as avoidance behavior or difficulties accepting the loss (see APA, 2013; [64], Shear et al., 2011). In the latest nosologies, a consensus was reached on the use of the term PGD (DSM-5-TR, APA, 2022; ICD-11, WHO2019). Accordingly, in the present systematic review, we apply this term broadly to encompass both earlier terminologies and instances where MGS scores reached clinically relevant thresholds. For children and adolescents, the time criterion of PGD is explicitly reduced from 12 to 6 months and the preoccupation may focus on the circumstances of death instead of the deceased person [5, 23]. However, Alvis and colleagues [4] proposed a further differentiation of the criteria for children and adolescents, in which the developmental stages are taken into account. Based on multidimensional grief theory, they emphasised that grief reactions in the areas of separation distress (e.g. developmental delay, anger), existential/identity distress (e.g. identity crises, fear of new attachments) and circumstance-related distress (e.g. stronger feelings of guilt due to lack of information about the death or the egocentric world view) can differ considerably from adults.

The conditional prevalence rates of PGD vary greatly between 10 and 32% depending on the loss (cf. [25]). Research has also demonstrated that prevalence rates of PGD doubled when the child or adolescent experienced the loss of a parent [25, 74]. The prevalence of PTSD is also significantly elevated in children and adolescents who have lost a relative—especially a parent—than in their peers who have not experienced loss (cf. [41]). While traumatic losses are considered to be particularly severe in the case of sudden deaths, there are also findings suggesting that protracted and foreseeable courses of illness can lead to higher prevalence rates than sudden traumatic losses due to the prolonged distress they cause [38].

In samples of adults, the interaction of the two disorders in their development and course has been widely documented (see systematic review by [37]). The results suggest that MGS/PGD may be a risk factor for later PTSS/PTSD rather than vice versa. There are isolated studies with children and adolescents that suggest similar correlations, i.e. that MGS/PGD and PTSS/PTSD predict each other in their development and maintenance [66].

Similarities were also found in the underlying risk factors when MGS/PGD and PTSS/PTSD were considered separately in samples of children and adolescents. To provide an appropriate overarching framework for comparing the risk factors of both phenomenologies, we follow previous reviews and meta-analyses and compare them within the overarching clusters of demographic, loss-related (pre-, peri-, post-loss), intrapersonal, and interpersonal factors (cf. [52, 75]).

Regarding demographic characteristics, numerous studies have shown that female gender is predictive of PTSD after traumatic events [75], but not after loss [45]. Female gender is also significantly associated with PGD [20, 51]. Although there are indications of other demographic risk factors such as age, socioeconomic status, or origin, the overall effect sizes are low and the influence of potential moderator variables is high [75]. There are also similarities regarding loss-related factors. Pre-existing mental illness and previous traumatic events are predictive of both PTSD [75] and PGD [20, 25] after loss. In a systematic review for adults, closeness to the deceased was found to be the greatest differentiating factor between PTSD and PGD [36]. In children and adolescents, the relationship to the deceased has been less frequently analysed and was found to be a decisive factor for both PGD [38, 74] and PTSD [21, 41]. The type of event and the severity of the trauma appear to be of considerable importance for PTSD [75]. The role of the type of loss is controversial with regard to the severity of PGD: prolonged and long-term death processes may be more stressful for children and adolescents than instances of sudden death [38]. On the other hand, Hubertus and Schneider [35] argue that progressive illnesses provide time for children to adapt to the new situation and develop appropriate coping strategies. Fundamentally, if circumstances of death are traumatic, the chance of developing mental disorders increases [3]. Peri-event, the degree of distress has been linked to PGD [66] and the level of anxiety and perceived life threat to PTSD (e.g., [46, 47]). Post-event, the way the child and the environment manage reactions to the event are of great importance and have a high effect size compared to other post-event risk factors [25, 75].

Internalising reactions such as cognitive avoidance [20] or self-attribution of guilt [55] are associated with PGD, while increased rumination and thought suppression are predictive of PTSD [71]. Low social support and feeling less loved and supported were also found to be significant risk factors for both disorders with regard to the reaction to the loss within the child or adolescent’s environment [49, 75], Alvis et al. 2023). In summary, similar drivers of both disorders have been identified, but there remains a troubling lack of research on differentiating risk factors for both disorders within the same sample.

In their systematic review, Jann and colleagues [36] sought to investigate possible discriminating factors between PTSD and PGD after traumatic loss in the same sample of adults and contribute to a prognosis of both disorders. The relationship to the deceased, mental health issues, and religious beliefs were identified as potentially specific risk factors for PGD. Social support and social emotions emerged as significant correlates and potential risk factors for both PTSD and PGD. Due to the long-term impairments for children and adolescents (cf. e.g. [13, 21]) and the developmental differences to adults (cf. e.g. [4]), different results are to be expected for children and potential risk factors. Therefore, the present study aims to address the identification of shared and differentiating risk factors, focusing on samples of children and adolescents. A key aspect is that, within the same samples, symptoms of MGS/PGD as well as PTSS/PTSD were assessed and analyzed, in order to enable differentiation between the two disorders despite their frequently overlap in risk factors. In view of the research situation described and considering the effect sizes, we hypothesize that intrapersonal and interpersonal reactions are more predictive of the development of MGS/PGD and PTSS/PTSD in children and adolescents than demographic characteristics and the objective circumstances of death. Due to the heterogenous study situation, no a priori hypothesis can be derived regarding risk factors differentiating between the two disorders.

Results may be used for prognostic purposes to identify high-risk groups within children and adolescents who would most benefit from receiving professional support following traumatic loss before symptoms manifest clinically.

## Methods

We preregistered the study protocol in PROSPERO in January 2024 (registration number: CRD42024497777). This review was conducted according to the criteria of the Preferred Reporting Items for Systematic Reviews and Meta-Analysis (PRISMA; Moher et al. 2009).

### Study inclusion

We included studies if (a) they investigated correlates of MGS and PTSS in the same sample, (b) based on the reported means and standard deviations, participants were on average 18 years of age or younger, (c) the children and adolescents experienced a loss in their immediate social context (d) at least parts of the sample experienced probable traumatic circumstances of death (e.g., homicide, suicide, accident, war, terror, natural disaster), (e) they used validated measurement instruments measuring MGS/PGD and PTSS/PTSD, (f) were published in English, and (g) were published in peer-reviewed journals.

We excluded studies in which (a) the circumstances of death were not reported, (b) the sample consisted exclusively of predictable, natural, or disease-related losses, (c) the losses were non-human (e.g., loss of a pet), and/or (d) the sample was mixed (e.g., adults and minors in the same sample and not considered separately in the analyses).

### Literature search strategy

We conducted the systematic literature search of the electronic databases Medline, PubMed, Web of Science, and APA PsycInfo in January 2026. A summary of the search strings used for each database is provided in Supplementary Materials A. For example, we used the following search string for PubMed: (traumatic loss OR suicide OR homicide OR accident OR natural disaster OR death) AND (child OR children OR child OR childhood trauma OR teenager OR adolescent OR adolescents OR kids OR boys OR girls OR pupil OR pupils OR student OR students OR orphan) AND (PTSD OR posttraumatic stress disorder OR posttraumatic stress OR traumatic stress OR stress disorder) AND (grief OR bereavement OR mourning OR PGD OR prolonged grief disorder OR PCBD OR persistent complex bereavement disorder OR CG OR complicated grief OR pathological grief OR traumatic grief OR traumatic bereavement) AND (predict* OR correlate* OR latent class analysis OR LCA OR regression).

We used the literature management programme Zotero to process the literature.

### Study selection and data extraction

The first, second, and fourth authors independently screened the titles and abstracts of all retrieved studies for potential eligibility. Afterwards, we conducted a full-text screening of the remaining studies, considering the inclusion and exclusion criteria (for a detailed list of exclusion reasons in full-text screening, see Supplementary Materials B). If there was disagreement about the eligibility of a study, we made the final decision involving the last author. We recorded the information contained in the publications using Table 1, which presents the following aspects: Study characteristics (title, authors, year, country of study), sample (sample size, age, gender), loss-related characteristics (cause of death, time since loss, relationship to the deceased), measurement instruments (including internal consistency), prevalence and potential risk factors for PGD, PTSD, and comorbidity. Risk factors that could potentially be assigned to multiple clusters were discussed in detail (e.g., functional impairment, whether it was primarily considered an intrapersonal characteristic or a symptom within the diagnostic of other disorders in the underlying study).

**Table 1:** Overview of included studies: sample characteristics, measures, prevalences, and risk factors

Sample No	Authors (year)	Country of sampling	Sample characteristics			Loss characteristics			Measures (α)		Probable prevalences			Potential risk factors		
			Size: n	Age: M (SD)	Female %	Cause of loss	Time since loss in years, M (SD)	Relationship to deceased	PTSS	MGS	PTSS	MGS	Comorbidity	PTSS	MGS	Comorbidity
1	Boelen and Spuij [9]	Netherlands	332	11.9 (2.9, 8–18)	56.9	Illness (55.2%), Violent (accident, suicide homicide 22.4%) unexpected medical cause (18.8%), other cause (3.6%)	2.71 (range: 1–119 months)	Mother, father, sibling, other relative	Child PTSD symptom scale (CPSS) (0.90)	Inventory of prolonged grief for children (IPG-C) (0.91) Inventory of prolonged grief for adolescents (IPG-A) (0.92)	51.5	n.a	n.a	Female gender Older age: 13–18 Higher depression severity Higher PGD severity Internalizing problems (parent-rated, CBCL) Total problems (CBCL)	PTSD symptom severity Functional impairment	
	Boelen et al. [12]	Netherlands	332	11.9 (2.9, 8–18)	56.9	Illness (55.2), Violent (accident, suicide, homocide 22.4%), unexpected medical cause (18.8%), other cause (3.6%)	2.71 (range: 1–119 months)	Mother, father, sibling, other relative	Child PTSD Symptom Scale (CPSS) (0.90)	Inventory of Prolonged Grief for Children (IPG-C) (0.91) Inventory of Prolonged Grief for Adolescents (IPG-A) (0.94)	n.a	35.2	26.2	n.a	Higher PTSD severity, (self-rated) o Higher depression severity (self-rated) o Functional impairment (self-rated)	Internalizing problems (parent-rated) Externalizing problems (parent-rated) Total problems (parent-rated) Bereavement-related PTSD Higher depression severity (self-rated) Functional impairment (self-rated)
	Spuij et al. [70]	Netherlands	332	9.9 (1.3); 8–12 14.9 (1.5); 13–18	49.7 (8–12 years) 67.4 (13–18 years)	Illness (53%), Violent (accident, suicide, homocide 24.4%), unexpected medical cause (17.3%), other (4.1%)	Children (age 8–12): 2.64 (S1) Adolescents (age 13–18): 2.83 (S2)	Mother, father, sibling, other relative	Child PTSD Symptom Scale (CPSS)children sample (0.88) and 0.66, respectively adolescent sample, (0.92) and (0.67), respectively)	Inventory of Prolonged Grief for Children (IPG-C) (0.91) and Inventory of Prolonged Grief for Adolescents (IPG-A) (0.94)	n.a	n.a	n.a	Older age (S2) Younger age (S1) Female gender (S2) Functional impairment	Older age (S2) Female gender (S2) Functional impairment	n.a
2	Boelen et al. [10]	Greek	t1: 213 t2: 137	14.83 (1.37, 13–18)	50.2	School bus accident	t1: 0.17 t2: 1.33	Peers	Children’s revised impact of events scale (CRIES-13) (t1: .73, t2: .82)	Traumatic grief inventory for children (TGIC) (t1: .87; t2: .91)	t1: 83.6 t2: 62.0	t1: 83.6 t2: 56.9	n.a	Traumatic grief symptom severity (TGIC)	PTSD avoidance symptom severity at t1 (CRIES-13)	Female gender Depression symptom severity (DSRS)
	Giannopoulou et al. [30]	Greek	168	14.5 (1.3, 12–17)	51.2	School bus accident	t1: 0.17 t2: 1.5	Peers	Children’s Revised Impact of Events Scale (CRIES-13) (0.75 at T1 and 0.83 at T2)	Traumatic Grief Inventory for Children (TGIC) (0.89 at T1 and 0.91 at T2)	t1: 78 t2: 55.1	t2: 21	n.a	direct event exposure (only short-term impact)	persisting high levels of posttraumatic stress symptoms (t2) persisting high levels of depression symptoms (t2) less social support (starting after 2 months) direct event exposure (only short-term impact)	n.a
3	Brown et al. [15]	USA	132	11.2 (2.7, 7–18)	48	medical (42%), interpersonal violence (31%), terrorism (10%), accident (5%) heart attacks, homicide, suicide, traffic accidents, and other accidents	1.73	parent 73%, sibling 36%, aunt/uncle 14%, grandparent 17%, close friend 4%, other (cousin) 29%	Child PTSD Symptom Scale (CPSS) (0.87)	Extended Grief Inventory (EGI) only TG subscale was used (0.94)	n.a	n.a	n.a	higher traumatic grief symptom severity higher depression symptom severity	closeness to deceased (parent vs. other relative) o child’s perceived life threat o short time since most difficult death o caregivers’ emotional reaction to death (e.g. anger, sadness), o degree to which people at home are sad o higher PTSD severity o higher depression symptom severity o child attended memorial service	n.a
4	Claycomb et al. [17]	Bosnia	1142	16.25 (1.07, 14–21)	70.0	Killed in the war and/or dying of unrelated causes to the war while war was happening	ca. 2 (post war, not time after individual loss)	Father (9%), mother (1%), sibling (4%), close relative (64%), close friend (51%)	UCLA PTSD Reaction Index (0.92, subscales used .66-.89)	Prototype approach mapping items from UCLA Grief Screening Scale (GSS) two distinct subscales Criterion B (0.67) and Criterion C (0.76)	n.a	n.a	n.a	o war related death (as opposed to natural/non-war causes) o experiencing both war-related and non-war related deaths (accumulation of loss) o prolonged grief symptom severity (esp. Criterion C—Reactive Distress and Social/Identity Disruption) o depression symptom severity	o age (older adolescent) o female gender o war related death (as opposed to natural/non-war causes) o experiencing both war-related and non-war related deaths (accumulation of loss) o PTSD symptom clusters o depression symptom severity	n.a
5	Dawson et al. [20]	Indonesia	110	10.43 (1.38, 7–13)	59.09	natural disaster (earthquake)	5.0	mothers, fathers, siblings, grandparents, uncle, cousin, close friends	Children's Revised Impact of Event Scale-13 (CRIES 13) (0.80)	Prolonged Grief Disorder 13-Child Version (PG-13) (n.a.)	48.0	24.0	n.a	o cognitive avoidance as coping o religious appraisal (higher beliefs of being protected)	o female gender o the total number of losses o cognitive avoidance as coping	n.a
6	Dodd et al. [22]	USA	726	12.52 (2.88, 6–18)	57.4	s1: natural disaster (49.5%), learning about or witnessing the violent death / injury of a loved one (49.5%) s2: learning about or witnessing the violent death or injury of a loved one (45.5%) and witnessing violence (19.3%)	n.a	parents, grandparent	UCLA PTSD Reaction Index (0.94)	Persistent Complex Bereavement Disorder (PCBD) Checklist, Criterion B and C scores (0.82) and (0.93) respectively	n.a	n.a	n.a	o higher suppression of emotions (AIS)	o higher suppression of emotions (AIS)	n.a
	Kassing et al. [43]	USA	583	12.16 (3.0, 7–18)	55.3	natural disaster, violent death, illness, accident, suicide, homicide, other death	n.a	loved one	UCLA PTSD Reaction Index for DSM-5 (0.94)	PCBD Checklist present study (0.82)	n.a	n.a	n.a	o polytrauma / polydeaths o ethnicity / race (identifying as Black) o older age	o gender (only if reason of death longterm-illness)	n.a
7	Giang et al. [29]	USA	197	12.36 (3.18, 7–21)	56.2	Sudden natural death (32.2%), long-term illness (32.3%), all other causes (not specified)	n.a	Loved one	UCLA PTSD Reaction Index (.92)	Persistent Complex Bereavement Disorder (PCBD) Checklist (Seperation Distress .9; Existential/Identity Distress .66 and Circumstance Related Distress .84)	n.a	n.a	n.a	o female gender o ethnicity/ race (identifying as black) o higher levels of future orientation o MGS symptom severity o depression symptom severity	o cause of death long-term illness for subscale Existential/ Identity Distress o PTSD symptom severity o depression symptom severity	n.a
8	Kaplow et al. [42]	USA	38	9.6 (2.04, 7–12)	52.6	sudden (40%), anticipated (34%), natural cause (i.e. disease), accident (13%), suicide (13%)	0.21	parents, adoptive parents	UCLA PTSD Reaction Index (0.91)	Inventory of Complicated Grief-Revised (ICG-R) (0.93)	n.a	n.a	n.a	o female gender o dampening of cortisol awakening response (day 1) o higher anxiety severity o higher maladaptive grief o avoidant coping o higher depression severity	o dampening of cortisol awakening response (day 1) o higher anxiety severity o higher PTSD severity o avoidant coping o higher depression severity	n.a
9	Lee et al. [49]	Korea	57	17.81 (0.44, 16–18)	0.49	Ferry disaster (100%)	1.6	Classmates	Child Report of Post-Traumatic Symptoms (CROPS)	Inventory for Complicated Grief (ICG)	26.3 (1,8)	24.5 (1,8)		n.a	o being an older student o PTSD symptom severity o lower score on autonomy and relationship with parents (KIDSCREEN-27)	n.a
10	McClatchey et al. [54]	USA	100	o range Camp A: 6–17 o range Camp B: 12–16	Camp A: 45.7 Camp B: 57.4	expected, sudden or violent	1.15 (range: 1–48 months)	mothers, fathers	UCLA PTSD (0.87)	Extended grief inventory (EGI) (0.92–0.93)	n.a	n.a	n.a	o delayed grief-treatment (no-short-term-treatment) o higher early symptoms (pretest scores)	o delayed grief-treatment (no-short-term treatment) o higher early symptoms (pretest scores)	n.a
11	Melhem et al. [57]	USA	182 (t1), 165 (t2), 141 (t3)	12,4 (2.8, 7–18)	45.6	Suicide (33,87), uninentional injury (25%), sudden natural death (41.13%)	t1: 0.7 (0.3) t2: 1.78 (0.35) t3: 2,76 (0.48)	Parents	Child PTSD Symptom Scale (CPSS)	Inventory for Complicated Grief (ICG-RC) (.95)	n.a	n.a	n.a	n.a	o previous history of depression o unintentional injury (accident) as cause of parent death o PTSD symptom severity (t1) o depression symptom severity (t1)	n.a
12	Özdemir et al. [61]	Turkey	205 (t1), 147 (t2)	13.22 (2.94, 8–17.5)	52.2	Earthquake (100%)	t1: 0.3/0.4 t2: 0.75	Parents	Children’s Revised Impact of Event Scale (CRIES-8) (.77)	Prolonged Grief Assessment–Child Version (PGA-C) (.92)	n.a	19.7 (t2)	n.a	o normative grief symptom severity (t1) o prolonged grief symptom severity (t2) o depressive symptom severity	o normative grief (t1) symptom severity o PTSD symptom severity o depressive symptom severity	
13	Poijula et al. [62]	Finland	89	15.4 (0.5, 14–17)	48.3	suicide	0.5	classmates (friends and not friends)	Impact of Event Scale (IES) (0.83)	Hogan Sibling Inventory of Bereavement (HSIB) (0.80)	n.a	n.a	n.a	o closeness to the deceased (friendship) o vieweing the body o participating funerals o combined closeness (close friendship, viewing the body, and participating in the funeral)	o perceived poor received support in intervention in school o combined closeness (close friendship, viewing the body, and participating in the funeral)	n.a
14	Revet et al. [66]	South West France	34	10.9 (3.2, 6–17)	67.6	Cancer (41.7%), suicide (25%), accident (20.8%), cardiac arrest (8.3%), genetic disease (4.2%)	t1: 0.30 t2: 0.64 t3: 1.07	mother, father	Peritraumatic Distress Inventory (PDI-C), Peritraumatic Dissociative Experiences Questionnaire (PDEQ-C) (n.a)	Inventory of complicated Grief–Revised for Children (ICG-RC) (0.95)	n.a	n.a	n.a	n.s	o higher peritraumatic distress	n.a

n/a = not applicable; n.s. = not significant

### Quality appraisal

The quality appraisal of the reviewed studies, conducted by the first and last author, was based on a valid instrument of the international research organisation JBI [58]. Final decisions were made after detailed examination and discussion of any discrepancies. As the inclusion criteria of the studies were narrow, we added the following specific questions to improve quality differentiation: "Is the sample size adequate?", "Were strategies to cope with confounding factors, especially covariates, reported?", "Were the results appropriately generalised considering the sample characteristics?" and "Was the study design longitudinal or cross-sectional?". The scaling ranged from 0–8 points, with 0–1 being rated as "poor", 2–3 as "below average", 4–5 as "average", 6–7 as "above average" and 8 as "excellent". We rated the studies independently of each other. Disagreements were discussed among all authors in order to reach a consensus.

## Results

**Fig. 1:**
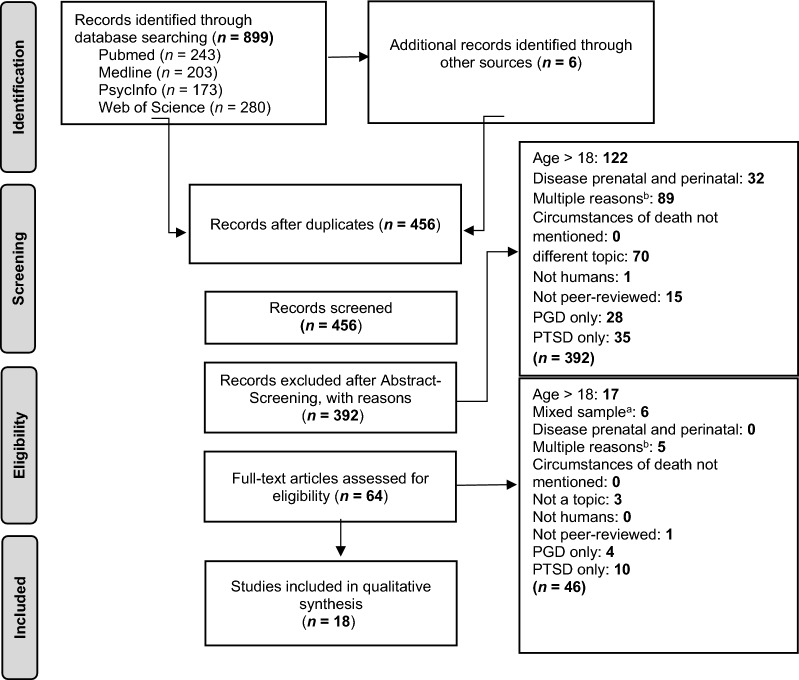
Flow chart of the selection process (PRISMA guidelines, Moher et al., 2009). ^a^Mixed sample = Participants with and without loss or adults and children mixed in the same sample, which were not separated in the analyses. ^b^Multiple reasons = Studies that were immediately recognisable as fulfilling several of the exclusion criteria

Using the above search strategy, the literature search yielded 905 potential articles, including 243 articles in Pubmed, 203 articles in Medline, 173 articles in PsycInfo, 280 articles in Web of Science, and 6 articles from other sources. After removing 449 duplicates, 456 articles remained for further screening. 392 articles were excluded during the title and abstract screening. Afterward, we performed a full-text screening of the remaining 64 studies. Finally, we identified 18 studies that met all inclusion criteria and no exclusion criteria. A detailed flow chart of this process is shown in Fig. 1.

### Study characteristics

The included studies were published between 2001 and 2025. The samples encompassing 5,034 individuals and were collected in the USA (*n* = 7), the Netherlands (*n* = 3), Greece (*n* = 2), Bosnia, Indonesia, Finland, France, Korea, and Turkey (*n* = 1 each). The age range was 6 to 21 years, with all studies reporting a mean age, including standard deviation, below 18 years (*M* = 12.88 years; *SD* = 2.19 years). Gender representation was mostly balanced, with the percentage of female participants ranging from 46 to 70% (*M* = 52%; *SD* = 14%). Three studies [9, 12, 70] were based on the same sample. Two further studies [10, 30] were also based on the same survey following a school bus accident. Dodd and colleagues [22] and Kassing and colleagues [43] also largely based their research on the same sample following a natural disaster. In the analysis, we therefore considered these studies together on the basis of the respective sample. Further information on the samples, loss characteristics, measurements, prevalences and potential risk factors can be found in Table 1.

With regard to the quality assessment, most of the studies were conducted satisfactorily. Four studies were rated as "excellent", 11 studies as "above average" and three studies as "average". Three of the studies had a longitudinal design, which was required for an excellent rating. Further information on the quality assessment can be found in Table 2.

**Table 2:** Quality Assessment of the included studies

Sample No		Were the criteria for inclusion in the sample clearly defined?	Were the study subjects and the setting described in detail?	Is the sample size adequate?	Were the outcomes measured in a valid and reliable way?	Were strategies to deal with confounding factors stated? Specially Covariates?	Was appropriate statistical analysis used?	Were findings adequately generalized considering sample characteristics?	Was the study design longitudinal or cross-sectional?	SUM	Excellent 8 Above Average 6–7 Average 4–5 Below Average 2–3 Poor 0–1
1	Boelen et al. [9]	1	1	1	1	1	1	1	0	7	Above Average
	Boelen et al. [12]	1	1	1	1	0	1	1	0	6	Above Average
	Spuij et al. [70]	1	1	1	1	0	1	1	0	6	Above Average
2	Boelen et al. [10]	1	1	1	1	1	1	1	1	8	Excellent
	Giannopoulou et al. [30]	1	1	1	1	1	1	1	1	8	Excellent
3	Brown et al. [15]	1	1	1	1	1	1	1	0	7	Above Average
4	Claycomb et al. [17]	1	1	1	0	1	1	1	0	6	Above Average
5	Dawson et al. [20]	1	0	1	1	0	1	0	0	4	Average
6	Dodd et al. [22]	0	1	1	1	0	1	1	0	5	Average
	Kassing et al. [43]	1	1	1	1	0	1	1	0	6	Above Average
7	Giang et al. [29]	1	0	1	1	1	1	1	0	6	Above Average
8	Kaplow et al. [42]	1	1	0	1	0	1	1	0	5	Average
9	Lee et al. [49]	1	1	1	1	1	1	1	0	7	Above Average
10	McClatchey et al. [54]	1	1	1	1	1	1	1	0	7	Above Average
11	Melhem et al. [57]	1	1	1	1	1	1	1	1	8	Excellent
12	Özdemir et al. [61]	1	1	1	1	1	1	1	1	8	Excellent
13	Poijula et al. [62]	1	1	1	0	1	1	1	0	6	Above Average
14	Revet et al. [66]	1	1	0	1	0	1	1	1	6	Above Average

### Loss-related characteristics

Based on the 14 samples assessed in the 18 studies, the loss events were mixed in eight samples (violent [e.g. accident, suicide, homicide], illness, or other causes), natural disaster in three samples, school bus accident, ferry disaster and suicide in one sample each.

The time since loss ranged from 1 month to 10 years. Almost half of the samples were surveyed within the first year. In the joint sample of Dodd and colleagues [22] and Kassing and colleagues [43], the time since loss could not be determined from the publications.

Regarding the relationship to the deceased, the 14 samples included either family connections (*n* = 11) or peers/classmates (*n* = 3).

### Measurement instruments

PTSS/PTSD were assessed using seven different validated measures. The University of California at Los Angeles Posttraumatic Stress Disorder Reaction Index (UCLA PTSD; [40, 72]) was used in five samples. The Children's Revised Impact of Events Scale (CRIES-13 [69]) was used in four samples, the Child PTSD Symptom Scale (CPSS [28]) was used in two samples. The Impact of Event Scale (IES, [34]), the Peritraumatic Distress Inventory (PDI-C [16]), the Peritraumatic Dissociative Experiences Questionnaire (PDEQ-C [16]), and the Child Report of Post-Traumatic Symptoms (CROPS [31]) were each used in individual studies.

MGS/PGD were assessed using eight different instruments. Four samples were examined using the Inventory of Complicated Grief—Revised for Children (ICG-RC; [56, 63]). The Extended Grief Inventory (EGI [14, 48]) and the Persistent Complex Bereavement Disorder Checklist (PCBD Checklist [39]) were applied twice. All other instruments were used in a single sample: Traumatic Grief Inventory for children (TGIC [77]), Prolonged Grief Disorder 13—Child version (PG-13 [65]), Inventory of Prolonged Grief—Childhood or—Adolescence (IPG-C/A [70]), Hogan Sibling Inventory of Bereavement (HSIB [33]), Prolonged Grief Assessment—Child Version (PGA-C [59]).

The instruments are based on different terminologies and constructs of MGS/PGD and PTSS/PTSD (for a comparison of the instruments, see Supplementary Materials C). However, both You and colleagues [80] and Boelen and Lenferink [8] demonstrated that the various inventories used to assess MGS/PGD and PTSS/PTSD generally show high convergent validity with one another, suggesting that they should all be capable of distinguishing normal from maladaptive symptomatology in line with current nosologies.

Furthermore, almost all samples were measured by adapted inventories for children and adolescence and showed good psychometric properties.

### Correlates and potential risk factors

**Fig. 2:**
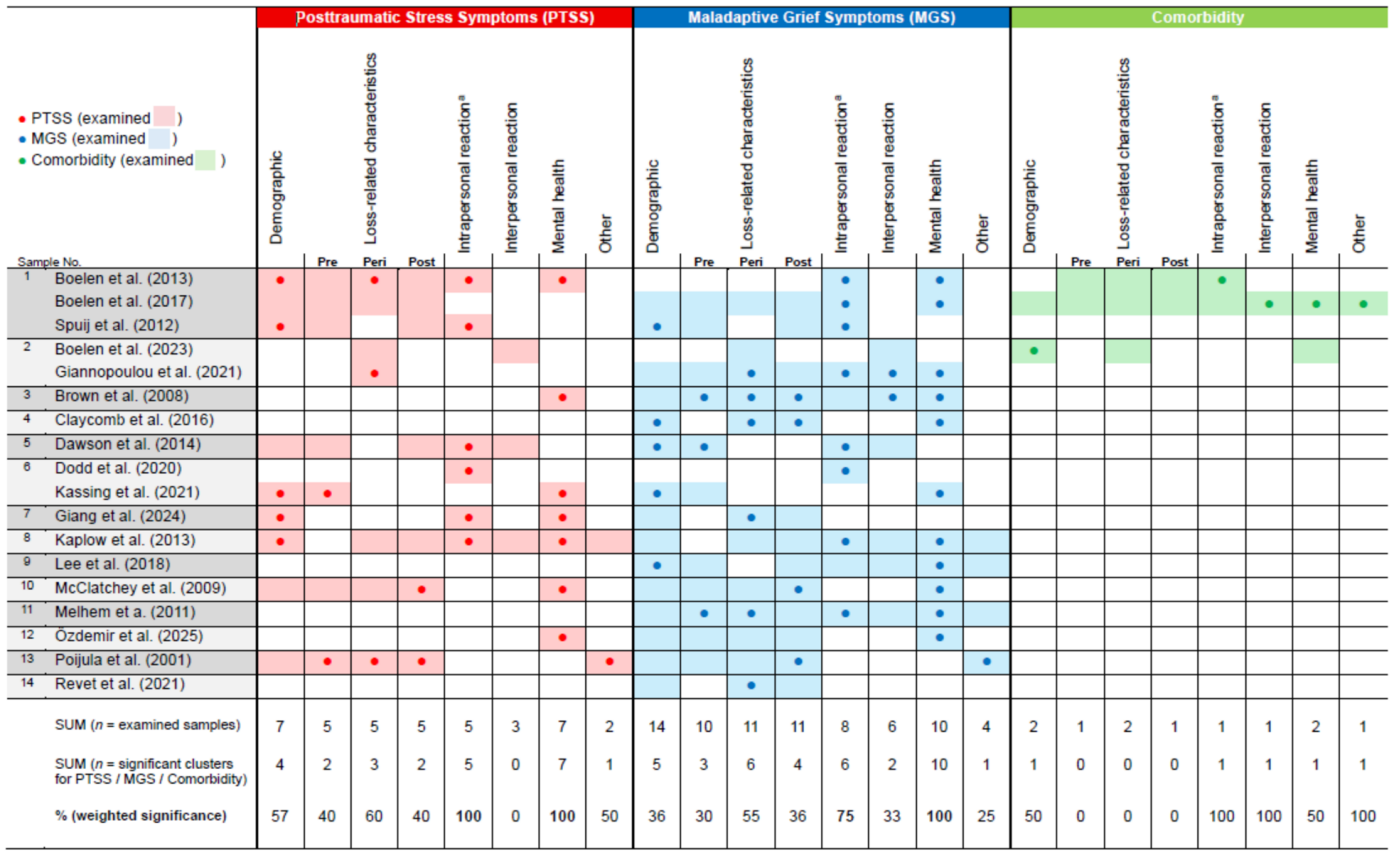
Overview of the analysed and significant predictors within the included studies. PTSS = posttraumatic stress symptoms; MGS = maladaptive grief symptoms; *N* = 18 studies based on *n* = 14 different samples were examined. Fields with a coloured background mean that the cluster was investigated for the respective disorder. If fields have a coloured dot, variables that were assigned to the respective cluster were significantly associated with the disorder. Relevance was assessed by weighting the number of significant clusters by the number in which the cluster was analysed. This was based on the number of different samples. ^a^For example, intrapersonal reactions showed a significant prediction for both MGS and PTSS in 75% and 100% of the studies with different samples

Due to the heterogeneity of the variables analysed, we grouped them into six superordinate clusters: Demographic characteristics, loss characteristics (pre-loss, peri-loss, post-loss), intrapersonal reaction, interpersonal reaction, other mental health impairments, and other. The specific variables of the respective studies we assigned to each cluster can be found in Table 3. To categorize which clusters were most frequently significantly associated with PTSS/PTSD, MGS/PGD, or comorbidity, we considered the total number of samples in which the respective cluster was investigated. The assigned risk factors were weighted based on the number of significant results relative to the number of studies examining the respective cluster regarding PTSS/PTSD, MGS/PGD, or comorbidity. The results are presented in Fig. 2.

**Table 3:**
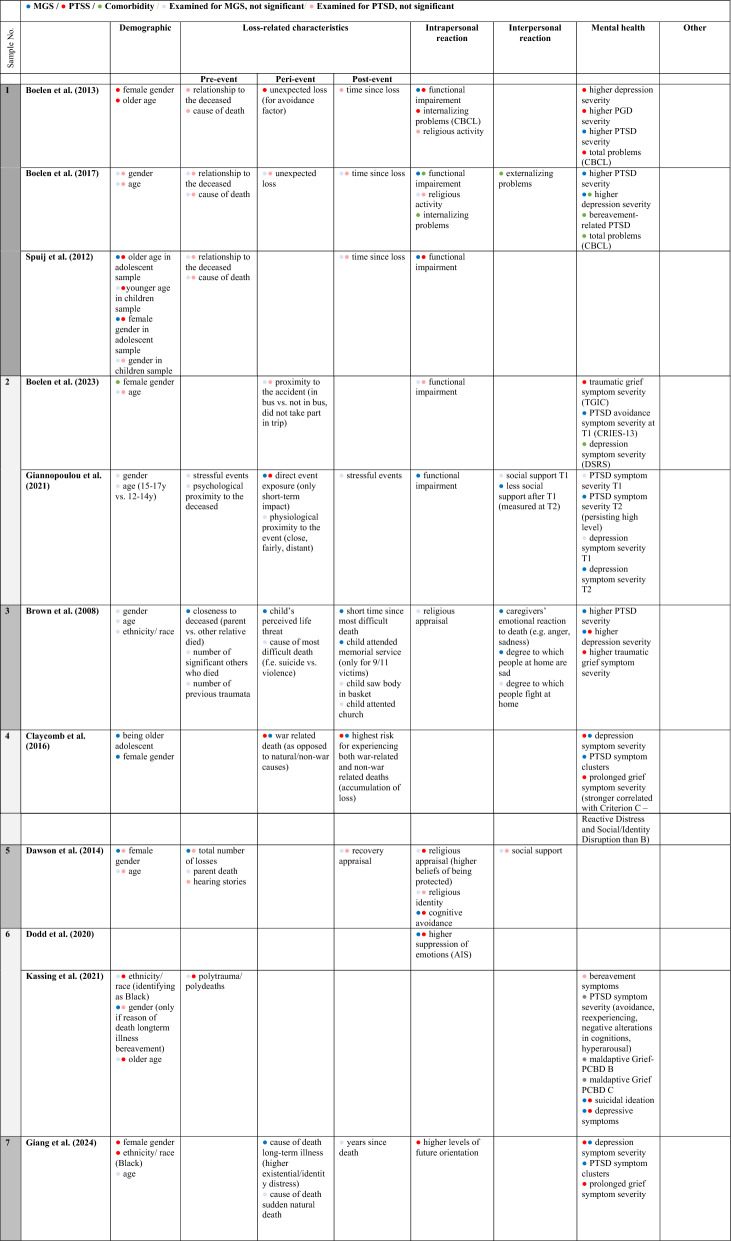

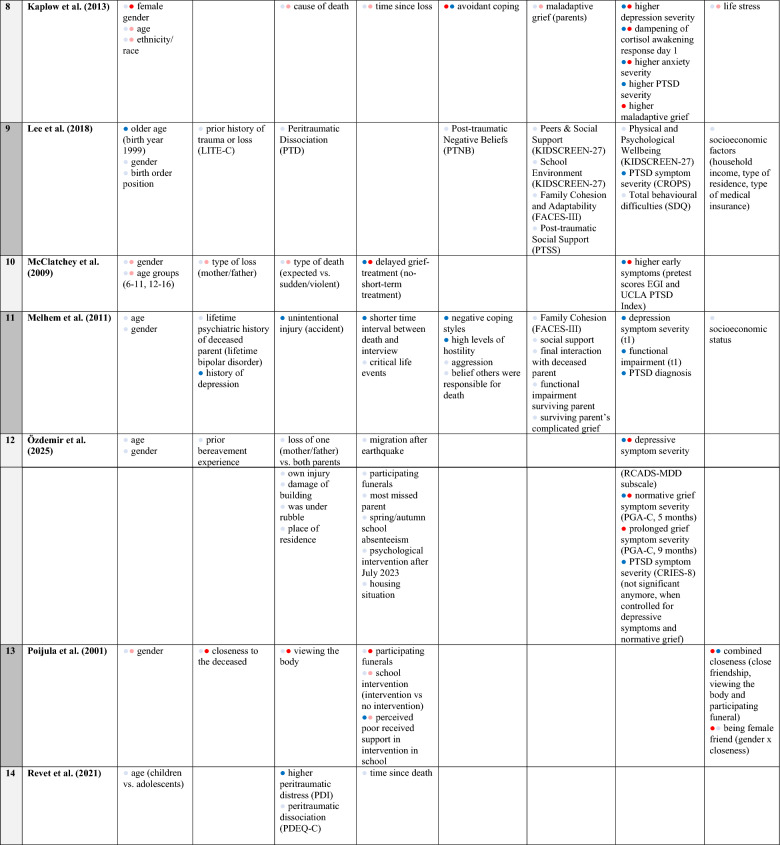
Overview of risk factors and their classification into six superordinate clusters (examined: significant and non-significant)

The cluster of intrapersonal reactions, which was analysed in five samples regarding PTSS/PTSD and eight samples regarding MGS/PGD, stood out for being significantly associated with both in 100% (PTSS/PTSD) and 75% (MGS/PGD) of the studies. Also striking was the mental health problems cluster, which was significantly associated with PTSS/PTSD and MGS/PGD in every sample where it was examined (100%). Peri- and post-loss characteristics were significant in 30–60% of the studies. Demographic characteristics and interpersonal reactions were least frequently associated with the respective disorders.

No valid statements can be made about correlates and risk factors for comorbidity, as these were only considered in three studies.

## Discussion

Loss in childhood and adolescence can trigger both MGS/PGD and PTSS/PTSD. This is the first systematic review to investigate which risk factors are shared between both phenomenologies or distinguish them from one another. Intrapersonal reactions are most frequently associated with MGS/PGD and PTSS/PTSD. In addition, other mental health problems were examined in most studies and were significantly associated with PTSS/PTSD and MGS/PGD in each of these studies, indicating a very high level of relevance. Demographic characteristics seem to play a minor role as risk factor. A conclusive assessment of other characteristics (e.g., traumatic circumstances of death) was not possible with the available data. The results should be interpreted with caution due to the small number of studies, their heterogeneity and cross-sectional designs. However, they may provide initial indications for the development of screening procedures to identify children and adolescents who are at a higher risk of psychopathological progression following a potentially traumatic loss.

Intrapersonal or internalising reactions were associated with both MGS/PGD and PTSS/PTSD in almost all studies and their underlying samples, highlighting their potential for psychopathological influences. On closer examination of the available data, the intrapersonal reactions were primarily functional impairment [9, 12, 70] as well as suppression of emotions and avoidance of emotions [22] and cognitions [20, 42]. Compared to risk factors that distinguish between MGS and PTSS in adults after traumatic loss, this finding represents a fundamental discrepancy (cf. [36]). The underlying studies share the commonality that all samples consist of the loss of a parent. The discrepancy in findings, particularly in relation to adults, could be attributed to the fact that children may suppress their own emotions and cognitions in order to prevent causing the grieving parent additional stress (cf., [79]). These avoidance strategies may become risk factors, as children try to hide their inner distress from the outside. This could also explain why intrapersonal risk factors are so prominent, while interpersonal factors appear to be less significant. Additionally, the surviving parent is often so immersed in their own grief that they may be unable to provide the necessary support for the child’s grieving process. Strover and colleagues [73] postulated that if parents fail to recognize the stress experienced by their children—whether due to their own grief or the child’s emotional suppression—they may not offer adequate coping support. This highlights a key reason why screening specifically for children and adolescents following the loss of a parent is crucial.

Although conclusions about the influence of pre-, peri- and post-death circumstances cannot be drawn, there is a tendency that the demographic characteristics of children and adolescents play a minor role in the development of disorders related to traumatic loss. This finding supports the idea that children and adolescents show universal reactions regardless of gender, age and origin, which are primarily determined by the internalisation of the event.

The presence of other mental health problems, particularly the respective other disorder, was a significant correlate of MGS/PGD and PTSS/PTSD in every single study examined. While the relationship and interaction between MGS, PTSS, depression and other mental illnesses has been replicated among adults (e.g. [50]), there is still much less research on comorbidity in children and adolescents. Initial studies provide evidence that, although the disorders differ in terms of symptoms, they frequently co-occur (e.g. [30]). However, the temporal relationships and how other mental health problems influence the development and maintenance of MGS/PGD and PTSS/PTSD cannot yet be evaluated at present.

### Limitations

To the best of our knowledge, this is the first systematic review to differentiate between PTSS/PTSD and MGS/PGD in children and adolescents. The quality of the underlying studies as well as the practical relevance for the development of screening procedures in the context of psychosocial crisis management are high.

On the other hand, the work impressively demonstrates that the field of research is still in its infancy and remains very limited with a small number of available studies. Firstly, both the small number of studies and, secondly, the predominantly cross-sectional design must be taken into account when interpreting the results. Thirdly, the heterogeneity of the underlying samples, particularly with regard to the circumstances of death (e.g., accident, disaster, suicide, illness), the age of the children and adolescents (6–21 years), and the time since the loss (1 months–10 years), also limits the clarity of the findings. The particularly wide range of prevalences seems to symbolise these restrictions. Fourthly, different inventories were used in the studies to assess PTSS/PTSD and MGS/PGD, including varying methods and case definitions. This heterogeneity complicates cross-study comparisons and reduces the reliability of conclusions about potential risk factors. Fifthly, the attempt to summarize the findings into categories of risk factors lacks robust methods to account for data heterogeneity, differences in study design, and potential confounding variables. This can lead to oversimplifications or inaccuracies in identifying actual risk factors versus correlates or consequences of the disorders. Sixthly, regarding generalizability, the diversity of survey countries should be taken into account, as cultural norms influence the expression of grief, rituals, and concepts of death. Cultural and societal factors in how children and families deal with death and grief cannot be ruled out. Although Aeschlimann and colleagues [1] identified a research trend toward considering cultural differences in grief processes in their scoping review, they also noted that the existing evidence remains highly heterogeneous, making it difficult to draw robust conclusions about the extent to which such differences may affect the presence or absence of risk factors. Finally, the current state of research in the area of PTSS/PTSD and MGS/PGD following loss in childhood and adolescents appears to be one of the greatest limitations. This harbours the danger that further important risk factors have not yet been identified due to the lack of a research base. Accordingly, given the current state of the evidence in this review, the absence of evidence (e.g., regarding demographic or loss-related variables) should not be equated with evidence of absence.

### Implications for research and practice

There is a fundamental need for a better understanding of PTSS/PTSD and MGS/PGD following loss in children and adolescents. An expansion of the study situation would help to better understand the relationships between loss-related variables (e.g. exposure to the death event, time since loss, relationship to the deceased, etc.) and the widely varying prevalence rates. The moderating effects of these variables would be of particular interest. In this context, development-related diagnostic criteria for PGD in children and adolescents are also urgently needed. The heterogeneity of existing instruments impairs the possibility of drawing precise conclusions in many respects. Future research should therefore utilise modern inventories that are based on the current ICD-11 and DSM-5-TR nosologies and take into account specific development-related reactions [23]. A good example would be the recently published Traumatic Grief Inventory for children [77]. Just as diagnostic instruments for age-appropriate quantification and assessment of PGD are still rare and at an early stage of development [4], there is currently a complete lack of screening procedures that can predict psychopathological processes following traumatic loss. Their possible practical relevance relates primarily to major events in the context of children and adolescents (e.g. school bus accidents, school shootings, etc.) in order to identify children and adolescents with a high-risk profile for psychopathological trajectories as early as possible. When developing such screening procedures, the question should also be addressed as to when it is appropriate to implement these tools following a traumatic loss. Finkeldei and colleagues [27] argue that children and adolescents need services that go beyond acute intervention to help them process their experiences. In the context of psychosocial crisis management, derived screening procedures would be the first empirical basis for predicting who would particularly need such support services and to provide these children or adolescents with the appropriate care. If future research confirms the hypothesis that children show intrapersonal reactions in order to avoid further burdening their social environment, such external services would be all the more important in supporting children and young people in their grief.

The finding that intrapersonal reactions in children and adolescents appeared to be particularly important in the prediction of PTSS/PTSD and MGS/PGD emphasises the need to address precisely these areas in interventions. For example, it is conceivable that the specific processing of feelings of guilt, the immediate reduction of avoidance behaviour or maintaining the level of functioning would be conducive to a healthy adaptation of children and adolescents to the loss event [78].

In this context, isolated studies have shown promising efficacy of trauma-focused cognitive behavioural therapy (TF-CBT) with additional grief modules in children and adolescents following traumatic loss [18, 19]. Unterhitzenberger and colleagues [76] also showed that TF-CBT (vs. waitlist) is highly effective in children and adolescents following traumatic loss in a randomised controlled trial (RCT). Taking into account the need for this research in conflict-affected areas, a study by O'Donnell and colleagues [60] gives hope, in which TF-CBT including grief modules was successfully implemented as group intervention in a Tanzanian orphanage. However, the research situation is still too limited to be able to make clear recommendations at this point.

From another perspective, Hiller and colleagues [32] have shown that parental behaviour that supports maladaptive adaption processes rather than counteracting them is associated with greater symptom severity in the longer term. In general, the behaviour of attachment figures, especially fathers, is considered to be of particular importance in coping [2, 47]. Focusing on intrapersonal reactions should therefore also be tested for its effectiveness in concepts such as ‘Parental Coaching’ [44] or ‘Child and Family Traumatic Stress Intervention’ [7].

In principle, it remains difficult to assess whether intrapersonal reactions are a risk factor for pathological progression or whether they result from the maladaptive grief reaction, as found by Melhem and colleagues [57]. More longitudinal studies starting shortly following the loss event are needed to illuminate any causal association.

## Conclusions

The loss of a loved one is among the most challenging and distressing life events that children and adolescents may face. Whether traumatic circumstances of loss increase the risk of psychopathological courses of MGS/PGD and PTSS/PTSD could not be definitively answered. However, further mental health problems and the intrapersonal reactions of children and adolescents were strongly correlated with both phenomenologies. Associated feelings of guilt, functional impairment and avoidance of thoughts and emotions should be taken into account in post-loss programmes (especially by parents). In addition, screening tools should particularly emphasise this symptom area when it comes to the question of which children and adolescents are more likely to develop MGS/PGD or PTSS/PTSD after a potentially traumatic loss. Psychosocial crisis management and the general psychological care structure would benefit enormously from further research into risk factors in this context. Further studies that collect data directly after the losses and have longitudinal designs are particularly necessary.

## Supplementary Information

Supplementary Material 1.

## Data Availability

Data available on request from the authors.
