# Supplementary material for: Risk factors for maladaptive grief and posttraumatic stress in children and adolescents following loss: a systematic review
Source: Child Adolesc Psychiatry Ment Health. 2026 Mar 29;20:55. doi: 10.1186/s13034-026-01076-7 (PMC13063894; doi:10.1186/s13034-026-01076-7)
Supplement: Supplementary file 1 — Supplementary Material 1. [file 13034_2026_1076_MOESM1_ESM.docx]

**SUPPLEMENTARY MATERIALS**

**Appendix A**

*Search Strings (Search conducted on 3^rd^ January 2026)*

**PubMed**

(traumatic loss OR suicide OR homicide OR accident OR natural disaster OR death) AND

(child OR children OR child OR childhood trauma OR teenager OR adolescent OR adolescents OR kids OR boys OR girls OR pupil OR pupils OR student OR students OR orphan)

AND (PTSD OR post traumatic stress disorder OR posttraumatic stress OR traumatic stress OR stress disorder)

AND (grief OR bereavement OR mourning OR PGD OR prolonged grief disorder OR PCBD OR persistent complex bereavement disorder OR CG OR complicated grief OR pathological grief OR traumatic grief OR traumatic bereavement)

AND (predict* OR correlate* OR latent class analysis OR LCA OR regression)

**APA Psychnet**

(Any Field: traumatic loss OR Any Field: suicide OR Any Field: homicide OR Any Field: accident OR Any Field: natural disaster) AND

(Any Field: child OR Any Field: children OR Any Field: childhood trauma OR Any Field: teenager OR Any Field: adolescent OR Any Field: adolescents OR Any Field: kids OR Any Field: boys OR Any Field: girls OR Any Field: pupil OR Any Field: pupils OR Any Field: student OR Any Field: students OR Any Field: orphan)

AND (Any Field: PTSD OR Any Field: post traumatic stress disorder OR Any Field: post traumatic stress OR Any Field: traumatic stress OR Any Field: stress disorder)

AND (Any Field: grief OR Any Field: bereavement OR Any Field: mourning OR Any Field: PGD OR Any Field: prolonged grief disorder OR Any Field: PCBD OR Any Field: persistent complex bereavement disorder OR Any Field: CG OR Any Field: complicated grief OR Any Field: pathological grief OR Any Field: traumatic grief OR Any Field: traumatic bereavement)

AND (Any Field: predict* OR Any Field: correlate* OR Any Field: latent class analysis OR Any Field: LCA OR Any Field: regression)

**Medline** (via KatalogPlus)

"(Alle Felder:traumatic loss ODER Alle Felder:suicide ODER Alle Felder:homicide ODER Alle Felder:accident ODER Alle Felder:natural disaster) UND (Alle Felder:child ODER Alle Felder:children ODER Alle Felder:childhood trauma ODER Alle Felder:teenager ODER Alle Felder:adolescent ODER Alle Felder:adolescents ODER Alle Felder:kids ODER Alle Felder:boys ODER Alle Felder:girls ODER Alle Felder:pupil ODER Alle Felder:pupils ODER Alle Felder:student ODER Alle Felder:students ODER Alle Felder:orphan) UND (Alle Felder:PTSD ODER Alle Felder:post traumatic stress disorder ODER Alle Felder:post traumatic stress ODER Alle Felder:traumatic stress ODER Alle Felder:stress disorder) UND (Alle Felder:grief ODER Alle Felder:bereavement ODER Alle Felder:mourning ODER Alle Felder:complex bereavement disorder ODER Alle Felder:CG ODER Alle Felder:complicated grief ODER Alle Felder:pathological grief ODER Alle Felder:traumatic grief ODER Alle Felder:traumatic bereavement) UND (Alle Felder:predict* ODER Alle Felder:correlate* ODER Alle Felder:latent class analysis ODER Alle Felder:LCA ODER Alle Felder:regression)"

**Medline via Ovid**

- *search different strings individually*

traumatic loss OR suicide OR homicide OR accident OR natural disaster OR death

child OR children OR child OR childhood trauma OR teenager OR adolescent OR adolescents OR kids OR boys OR girls OR pupil OR pupils OR student OR students OR orphan

PTSD OR post traumatic stress disorder OR posttraumatic stress OR traumatic stress OR stress disorder

grief OR bereavement OR mourning OR PGD OR prolonged grief disorder OR PCBD OR persistent complex bereavement disorder OR CG OR complicated grief OR pathological grief OR traumatic grief OR traumatic bereavement

grief OR bereavement OR mourning OR PGD OR prolonged grief disorder OR PCBD OR persistent complex bereavement disorder OR CG OR complicated grief OR pathological grief OR traumatic grief OR traumatic bereavement

predict* OR correlate* OR latent class analysis OR LCA OR regression

- *connect the different search strings manually with AND in search history*

**Web of Science**

- *search different strings individually*

ALL = (traumatic loss OR suicide OR homicide OR accident OR natural disaster OR death)

ALL = (child OR children OR child OR childhood trauma OR teenager OR adolescent OR adolescents OR kids OR boys OR girls OR pupil OR pupils OR student OR students OR orphan)

ALL = (PTSD OR post traumatic stress disorder OR posttraumatic stress OR traumatic stress OR stress disorder)

ALL = (grief OR bereavement OR mourning OR PGD OR prolonged grief disorder OR PCBD OR persistent complex bereavement disorder OR CG OR complicated grief OR pathological grief OR traumatic grief OR traumatic bereavement)

ALL = (grief OR bereavement OR mourning OR PGD OR prolonged grief disorder OR PCBD OR persistent complex bereavement disorder OR CG OR complicated grief OR pathological grief OR traumatic grief OR traumatic bereavement)

ALL = (predict* OR correlate* OR latent class analysis OR LCA OR regression)

*connect the different search strings manually with AND in search history*

**Appendix B**

*List of articles and reasons for exclusion from full-text screening:*

| **Article** | **Reason of exclusion** |
| --- | --- |
| Azoulay, E., Resche-Rigon, M., Megarbane, B., Reuter, D., Labbé, V., Cariou, A., Géri, G., Van der Meersch, G., Kouatchet, A., Guisset, O., Bruneel, F., Reignier, J., Souppart, V., Barbier, F., Argaud, L., Quenot, J.-P., Papazian, L., Guidet, B., Thiéry, G., … Kentish-Barnes, N. (2022). Association of COVID-19 Acute Respiratory Distress Syndrome With Symptoms of Posttraumatic Stress Disorder in Family Members After ICU Discharge. JAMA, 327(11), 1042–1050. https://doi.org/10.1001/jama.2022.2017 | only PTSD-measurement |
| Barthes, A., Razafimandimby-Haelewyn, A., & Bui, E. (2021). Prolonged grief disorder in the postpartum: An editorial. International Journal of Mental Health, 50(4), 290–292. APA PsycInfo. https://doi.org/10.1080/00207411.2021.1997135 | no statistical analysis |
| Beauquier-Maccotta, B., Shulz, J., De Wailly, D., Meriot, M.-E., Soubieux, M.-J., Ouss, L., Grosmaitre, C., Salomon, L. J., Golse, B., Ville, Y., & Missonnier, S. (2022). Prenatal attachment, anxiety and grief during subsequent pregnancy after medical termination of pregnancy. Attachment to which child? JOURNAL OF GYNECOLOGY OBSTETRICS AND HUMAN REPRODUCTION, 51(4). https://doi.org/10.1016/j.jogoh.2022.102353 | parents / adults in context of pre-/peri-/post-natal loss |
| Bennett, S. M., Litz, B. T., Maguen, S., & Ehrenreich, J. T. (2008). An Exploratory Study of the Psychological Impact and Clinical Care of Perinatal Loss. JOURNAL OF LOSS & TRAUMA, 13(6), 485–510. https://doi.org/10.1080/15325020802171268 | parents / adults in context of pre-/peri-/post-natal loss |
| Chan, C. M. H., Ng, C. G., Taib, N. A., Wee, L. H., Krupat, E., & Meyer, F. (2018). Course and Predictors of Post-Traumatic Stress Disorder in a Cohort of Psychologically Distressed Patients With Cancer: A 4-Year Follow-Up Study. CANCER, 124(2), 406–416. https://doi.org/10.1002/cncr.30980 | adults |
| Christiansen, D. M., Elklit, A., & Olff, M. (2013). Parents bereaved by infant death: PTSD symptoms up to 18 years after the loss. General Hospital Psychiatry, 35(6), 605–611. https://doi.org/10.1016/j.genhosppsych.2013.06.006 | parents / adults in context of pre-/peri-/post-natal loss |
| Christiansen, D. M., Olff, M., & Elklit, A. (2014). Parents bereaved by infant death: Sex differences and moderation in PTSD, attachment, coping and social support. General Hospital Psychiatry, 36(6), 655–661. https://doi.org/10.1016/j.genhosppsych.2014.07.012 | parents / adults in context of pre-/peri-/post-natal loss |
| Clark, O. E., Fortney, C. A., Dunnells, Z. D. O., Gerhardt, C. A., & Baughcum, A. E. (2021). Parent Perceptions of Infant Symptoms and Suffering and Associations With Distress Among Bereaved Parents in the NICU. Journal of Pain and Symptom Management, 62(3), e20–e27. https://doi.org/10.1016/j.jpainsymman.2021.02.015 | parents / adults in context of pre-/peri-/post-natal loss |
| Cowchock, F. S., Ellestad, S. E., Meador, K. G., Koenig, H. G., Hooten, E. G., & Swamy, G. K. (2011). Religiosity is an important part of coping with grief in pregnancy after a traumatic second trimester loss. Journal of Religion and Health, 50(4), 901–910. https://doi.org/10.1007/s10943-011-9528-y | parents / adults in context of pre-/peri-/post-natal loss |
| Devylder, J. E., Wang, J. S.-H., Oh, H. Y., & Lukens, E. P. (2013). Child loss and psychosis onset: Evidence for traumatic experience as an etiological factor in psychosis. Psychiatry Research, 205(1–2), 90–94. https://doi.org/10.1016/j.psychres.2012.08.008 | no PTSD- and grief measurement |
| Dumont, E., Bourque, C. J., Duval, M., Payot, A., Sultan, S., & Team, B. P. R. (2020). A Portrait of Self-Reported Health and Distress in Parents Whose Child Died of Cancer. OMEGA-JOURNAL OF DEATH AND DYING. https://doi.org/10.1177/0030222820959943 | parents / adults in context child’s loss |
| Dupont, T., Kentish-Barnes, N., Pochard, F., Duchesnay, E., & Azoulay, E. (2023). Prediction of post-traumatic stress disorder in family members of ICU patients: A machine learning approach. Intensive Care Medicine. https://doi.org/10.1007/s00134-023-07288-1 | adults / only PTSD-measurement |
| Dyregrov, K. (2004). Bereaved parents’ experience of research participation. Social Science & Medicine (1982), 58(2), 391–400. https://doi.org/10.1016/s0277-9536(03)00205-3 | parents / adults in context of pre-/peri-/post-natal loss |
| Elklit, A., Reinholt, N., Nielsen, L. H., Blum, A., & Lasgaard, M. (2010). Posttraumatic stress disorder among bereaved relatives of cancer patients. Journal of Psychosocial Oncology, 28(4), 399–412. https://doi.org/10.1080/07347332.2010.488142 | adults / only PTSD-measurement |
| Fernandez-Alcantara, M., Nieves Perez-Marfil, M., Catena-Martinez, A., & Cruz-Quintana, F. (2017). Grieving and loss processes: Latest findings and complexities. ESTUDIOS DE PSICOLOGIA, 38(3, SI), 561–581. https://doi.org/10.1080/02109395.2017.1328210 | no statistical analysis |
| Fisher, J. E., Rice, A. J., Zuleta, R. F., & Cozza, S. J. (2022). Bereavement during the COVID-19 Pandemic: Impact on Coping Strategies and Mental Health. PSYCHIATRY-INTERPERSONAL AND BIOLOGICAL PROCESSES, 85(4), 354–372. https://doi.org/10.1080/00332747.2022.2051141 | no PTSD-measurement |
| Guclu, O., Senormanci, G., Tuten, A., Gok, K., & Senormanci, O. (2021). Perinatal Grief and Related Factors After Termination of Pregnancy for Fetal Anomaly: One-Year Follow-up Study. NOROPSIKIYATRI ARSIVI-ARCHIVES OF NEUROPSYCHIATRY, 58(3), 221–227. https://doi.org/10.29399/npa.25110 | parents / adults in context of pre-/peri-/post-natal loss |
| Hamdan, S., Melhem, N. M., Porta, G., Walker Payne, M., & Brent, D. A. (2012). The phenomenology and course of depression in parentally bereaved and non-bereaved youth. Journal of the American Academy of Child and Adolescent Psychiatry, 51(5), 528–536. https://doi.org/10.1016/j.jaac.2012.02.021 | parents / adults in context of pre-/peri-/post-natal loss |
| Hennegan, J. M., Henderson, J., & Redshaw, M. (2018). Is partners’ mental health and well-being affected by holding the baby after stillbirth? Mothers’ accounts from a national survey. Journal of Reproductive and Infant Psychology, 36(2), 120–131. https://doi.org/10.1080/02646838.2018.1424325 | parents / adults in context of pre-/peri-/post-natal loss |
| Horsch, A., Jacobs, I., & McKenzie-McHarg, K. (2015). Cognitive Predictors and Risk Factors of PTSD Following Stillbirth: A Short-Term Longitudinal Study. JOURNAL OF TRAUMATIC STRESS, 28(2), 110–117. https://doi.org/10.1002/jts.21997 | parents / adults in context of pre-/peri-/post-natal loss |
| Howell, K. H., Barrett-Becker, E. P., Burnside, A. N., Wamser-Nanney, R., Layne, C. M., & Kaplow, J. B. (2016). Children Facing Parental Cancer Versus Parental Death: The Buffering Effects of Positive Parenting and Emotional Expression. JOURNAL OF CHILD AND FAMILY STUDIES, 25(1), 152–164. https://doi.org/10.1007/s10826-015-0198-3 | no grief-measurement |
| Huberty, J., Sullivan, M., Green, J., Kurka, J., Leiferman, J., Gold, K., & Cacciatore, J. (2020). Online yoga to reduce post traumatic stress in women who have experienced stillbirth: A randomized control feasibility trial. BMC Complementary Medicine and Therapies, 20(1), 173. https://doi.org/10.1186/s12906-020-02926-3 | parents / adults in context of pre-/peri-/post-natal loss |
| Hunfeld, J. A., Wladimiroff, J. W., & Passchier, J. (1997). Prediction and course of grief four years after perinatal loss due to congenital anomalies: A follow-up study. The British Journal of Medical Psychology, 70 ( Pt 1), 85–91. https://doi.org/10.1111/j.2044-8341.1997.tb01888.x | parents / adults in context of pre-/peri-/post-natal loss |
| Kersting, A., Kroker, K., Steinhard, J., Lüdorff, K., Wesselmann, U., Ohrmann, P., Arolt, V., & Suslow, T. (2007). Complicated grief after traumatic loss: A 14-month follow up study. European Archives of Psychiatry and Clinical Neuroscience, 257(8), 437–443. https://doi.org/10.1007/s00406-007-0743-1 | Adults |
| Kinser, P. A., Jallo, N., Amstadter, A. B., Thacker, L. R., Jones, E., Moyer, S., Rider, A., Karjane, N., & Salisbury, A. L. (2021). Depression, Anxiety, Resilience, and Coping: The Experience of Pregnant and New Mothers During the First Few Months of the COVID-19 Pandemic. JOURNAL OF WOMENS HEALTH, 30(5), 654–664. https://doi.org/10.1089/jwh.2020.8866 | parents / adults in context of pre-/peri-/post-natal loss |
| Krosch, D. J., & Shakespeare-Finch, J. (2017). Grief, traumatic stress, and posttraumatic growth in women who have experienced pregnancy loss. Psychological Trauma: Theory, Research, Practice and Policy, 9(4), 425–433. https://doi.org/10.1037/tra0000183 | parents / adults in context of pre-/peri-/post-natal loss |
| Kroth, J., Garcia, M., Hallgren, M., LeGrue, E., Ross, M., & Scalise, J. (2004). Perinatal loss, trauma, and dream reports. Psychological Reports, 94(3 Pt 1), 877–882. https://doi.org/10.2466/pr0.94.3.877-882 | parents / adults in context of pre-/peri-/post-natal loss |
| Kukulskienė, M., & Žemaitienė, N. (2022). Postnatal Depression and Post-Traumatic Stress Risk Following Miscarriage. International Journal of Environmental Research and Public Health, 19(11), 6515. https://doi.org/10.3390/ijerph19116515 | parents / adults in context of pre-/peri-/post-natal loss |
| Leithner, K., Maar, A., Fischer-Kern, M., Hilger, E., Löffler-Stastka, H., & Ponocny-Seliger, E. (2004). Affective state of women following a prenatal diagnosis: Predictors of a negative psychological outcome. ULTRASOUND IN OBSTETRICS & GYNECOLOGY, 23(3), 240–246. https://doi.org/10.1002/uog.978 | parents / adults in context of pre-/peri-/post-natal loss |
| Li, J., Olsen, J., Vestergaard, M., & Obel, C. (2010). Attention-deficit/hyperactivity disorder in the offspring following prenatal maternal bereavement: A nationwide follow-up study in Denmark. European Child & Adolescent Psychiatry, 19(10), 747–753. https://doi.org/10.1007/s00787-010-0113-9 | parents / adults in context of pre-/peri-/post-natal loss |
| Li, J., Vestergaard, M., Obel, C., Christensen, J., Precht, D. H., Lu, M., & Olsen, J. (2009). A nationwide study on the risk of autism after prenatal stress exposure to maternal bereavement. Pediatrics, 123(4), 1102–1107. https://doi.org/10.1542/peds.2008-1734 | parents / adults in context of pre-/peri-/post-natal loss |
| Lowe, S. R., Manove, E. E., & Rhodes, J. E. (2013). Posttraumatic Stress and Posttraumatic Growth Among Low-Income Mothers Who Survived Hurricane Katrina. JOURNAL OF CONSULTING AND CLINICAL PSYCHOLOGY, 81(5), 877–889. https://doi.org/10.1037/a0033252 | adults / mothers |
| Murphy, S., Shevlin, M., & Elklit, A. (2014). Psychological Consequences of Pregnancy Loss and Infant Death in a Sample of Bereaved Parents. JOURNAL OF LOSS & TRAUMA, 19(1), 56–69. https://doi.org/10.1080/15325024.2012.735531 | parents / adults in context of pre-/peri-/post-natal loss |
| Reitsma, L., Boelen, P. A., de Keijser, J., & Lenferink, L. I. M. (2021). Online treatment of persistent complex bereavement disorder, posttraumatic stress disorder, and depression symptoms in people who lost loved ones during the COVID-19 pandemic: Study protocol for a randomized controlled trial and a controlled trial. EUROPEAN JOURNAL OF PSYCHOTRAUMATOLOGY, 12(1). https://doi.org/10.1080/20008198.2021.1987687 | adults |
| Robinson, G. E. (2014). Pregnancy loss. BEST PRACTICE & RESEARCH CLINICAL OBSTETRICS & GYNAECOLOGY, 28(1), 169–178. https://doi.org/10.1016/j.bpobgyn.2013.08.012 | parents / adults in context of pre-/peri-/post-natal loss |
| Ryninks, K., Wilkinson-Tough, M., Stacey, S., & Horsch, A. (2022). Comparing posttraumatic growth in mothers after stillbirth or early miscarriage. PloS One, 17(8), e0271314. https://doi.org/10.1371/journal.pone.0271314 | parents / adults in context of pre-/peri-/post-natal loss |
| Scheidt, C. E., Hasenburg, A., Kunze, M., Waller, E., Pfeifer, R., Zimmermann, P., Hartmann, A., & Waller, N. (2012). Are individual differences of attachment predicting bereavement outcome after perinatal loss? A prospective cohort study. Journal of Psychosomatic Research, 73(5), 375–382. https://doi.org/10.1016/j.jpsychores.2012.08.017 | parents / adults in context of pre-/peri-/post-natal loss |
| Snaman, J. M., Mazzola, E., Helton, G., Feifer, D., Morris, S. E., Clark, L., Baker, J. N., & Wolfe, J. (2023). Early Bereavement Psychosocial Outcomes in Parents of Children Who Died of Cancer With a Focus on Social Functioning. JCO Oncology Practice, 19(4), e527–e541. https://doi.org/10.1200/OP.22.00538 | parents / adults in context of child’s loss |
| Su, X., Liang, H., Yuan, W., Olsen, J., Cnattingius, S., & Li, J. (2016). Prenatal and early life stress and risk of eating disorders in adolescent girls and young women. EUROPEAN CHILD & ADOLESCENT PSYCHIATRY, 25(11), 1245–1253. https://doi.org/10.1007/s00787-016-0848-z | parents / adults in context of pre-/peri-/post-natal loss |
| Su, X., Yu, Y., Meng, L., Duan, T., Zhao, Y., Laszlo, K. D., Valdimarsdottir, U. A., Hua, J., & Li, J. (2021). Prenatal Maternal Bereavement and Its Association With Intellectual Disability in the Offspring. PSYCHOSOMATIC MEDICINE, 83(8), 887–893. https://doi.org/10.1097/PSY.0000000000000990 | parents / adults in context of pre-/peri-/post-natal loss |
| Tafa, M., Cerniglia, L., Cimino, S., Ballarotto, G., Marzilli, E., & Tambelli, R. (2018). Predictive Values of Early Parental Loss and Psychopathological Risk for Physical Problems in Early Adolescents. FRONTIERS IN PSYCHOLOGY, 9. https://doi.org/10.3389/fpsyg.2018.00922 | parents / adults in context of pre-/peri-/post-natal loss |
| Tang, S., & Xiang, Z. (2021). Who suffered most after deaths due to COVID-19? Prevalence and correlates of prolonged grief disorder in COVID-19 related bereaved adults. GLOBALIZATION AND HEALTH, 17(1). https://doi.org/10.1186/s12992-021-00669-5 | no PTSD-measurement |
| Turton, P., Hughes, P., Evans, C. D., & Fainman, D. (2001). Incidence, correlates and predictors of post-traumatic stress disorder in the pregnancy after stillbirth. The British Journal of Psychiatry: The Journal of Mental Science, 178, 556–560. https://doi.org/10.1192/bjp.178.6.556 | parents / adults in context of pre-/peri-/post-natal loss |
| Turton, P., Hughes, P., Fonagy, P., & Fainman, D. (2004). An investigation into the possible overlap between PTSD and unresolved responses following stillbirth: An absence of linkage with only unresolved status predicting infant disorganization. Attachment & Human Development, 6(3), 241–253. APA PsycInfo. https://doi.org/10.1080/14616730412331281575 | parents / adults in context of pre-/peri-/post-natal loss |
| Unal, Y., Unal, Y., Ozturk, D. A., Emir, G. K., Yilmaz, M., & Kutlu, G. (2017). The Evaluation Acute Traumatic Stress Level in Close Relatives of Stroke Patients. PSYCHIATRY INVESTIGATION, 14(5), 546–549. https://doi.org/10.4306/pi.2017.14.5.546 | adults |
| Uren, T., & Wastell, C. (2002). Attachment and meaning-making in perinatal bereavement. DEATH STUDIES, 26(4), 279–308. https://doi.org/10.1080/074811802753594682 | parents / adults in context of pre-/peri-/post-natal loss |
| Wellisch, D. K., Ormseth, S. R., Hartoonian, N., & Owen, J. E. (2012). A retrospective study predicting psychological vulnerability in adult daughters of breast cancer patients. Families, Systems & Health: The Journal of Collaborative Family Healthcare, 30(3), 253–264. https://doi.org/10.1037/a0029638 | no loss |
| Zetumer, S., Young, I., Shear, M. K., Skritskaya, N., Lebowitz, B., Simon, N., Reynolds, C., Mauro, C., & Zisook, S. (2015). The impact of losing a child on the clinical presentation of complicated grief. JOURNAL OF AFFECTIVE DISORDERS, 170, 15–21. https://doi.org/10.1016/j.jad.2014.08.021 | parents / adults in context of pre-/peri-/post-natal loss |
|  |  |
|  |  |

**Appendix C**

**Table C.1**

Comparison of measuring instruments for MGS/PGD

| **Inventory** | **Authors** | **Description** |
| --- | --- | --- |
| Inventory of Complicated Grief - Revised for Children (ICG-RC) | Prigerson et al., 1995; Melhem et al., 2013 | - adaptation of the adult Inventory of Complicated Grief for use with children and adolescents. - assesses core symptoms of complicated grief, such as intense yearning, difficulty accepting the death, emotional pain, and preoccupation with the deceased. - reflect the severity of maladaptive grief reactions associated with functional impairment. - goal is to identify children at risk for persistent, clinically significant grief responses. |
| Extended Grief Inventory (EGI) | Brown & Goodman, 2005; Layne et al., 2001) | - broad range of grief reactions in children and adolescents. - assesses multiple dimensions of grief, including traumatic grief symptoms, ongoing emotional presence of the deceased, and positive memories. - goal is to provide a comprehensive assessment of grief responses, not limited to diagnostic thresholds. |
| Persistent Complex Bereavement Disorder Checklist (PCBD Checklist) | Kaplow et al., 2018 | - operationalizes the DSM-5 proposed criteria for Persistent Complex Bereavement Disorder - assesses separation distress, reactive distress, identity disruption, and associated functional impairment. - goal is diagnostic screening and clinical characterization of PCBD symptomatology in children and adolescents. |
| Traumatic Grief Inventory for children (TGIC) | Van Dijk et al., 2023 | - focusses on grief reactions in children with a specific focus on trauma-related aspects of bereavement. - assesses intrusive thoughts, emotional and physiological reactions to reminders, and difficulties integrating the loss. - goal is to measure traumatic grief severity rather than general bereavement reactions. |
| Prolonged Grief Disorder 13 - Child version (PG-13) | Prigerson et al., 2008 | - core symptoms of Prolonged Grief Disorder - assesses persistent yearning, preoccupation with the deceased, emotional pain, and functional impairment over time. - goal is brief, standardized assessment of PGD symptom criteria. |
| Inventory of Prolonged Grief - Childhood or - Adolescence (IPG-C/A) | Spuij et al., 2012 | - two age-specific versions: IPG-C (children) and IPG-A (adolescents), each containing 30 items. - assesses e.g. intense longing, emotional numbness, avoidance, and difficulty moving forward. - goal is to identify persistent and maladaptive grief reactions in youth. |
| Hogan Sibling Inventory of Bereavement (HSIB) | Hogan, 1990 | - bereavement reactions specifically in siblings following the death of a brother or sister. - assesses emotional, social, and behavioral responses, including changes in relationships and personal development. - goal is to provide a multidimensional understanding of sibling bereavement experiences. |
| Prolonged Grief Assessment - Child Version (PGA-C) | Nader & Prigerson, 2020 | - tool for symptoms of Prolonged Grief Disorder. - assesses core PGD features such as persistent yearning, difficulty accepting the death, emotional distress, and functional impairment. - goal is clinical assessment of prolonged grief severity in children. |

**Table C.2**

Comparison of measuring instruments for PTSS/PTSD

| **Inventory** | **Authors** | **Description** |
| --- | --- | --- |
| University of California at Los Angeles Posttraumatic Stress Disorder Reaction Index (UCLA PTSD) | Kaplow et al., 2020; Steinberg et al., 2004 | - posttraumatic stress symptoms in children and adolescents following exposure to traumatic events (DSM-5). - assesses intrusion, avoidance, negative alterations in cognition and mood, and hyperarousal. - goal is standardized assessment of PTSD symptom severity and probable diagnosis in youth. |
| Children's Revised Impact of Events Scale (CRIES-13) | Smith et al., 2003 | - posttraumatic stress symptoms in children aged approximately 8 years and older. - assesses intrusion, avoidance, and hyperarousal. - goal is a screening rather than a full diagnostic assessment. |
| Child PTSD Symptom Scale (CPSS) | Foa et al., 2001 | - posttraumatic stress symptoms in children and adolescents (DSM-IV). - assesses intrusion, avoidance, hyperarousal, and associated functional impairment. - goal is to assess PTSD symptom presence, severity, and impact on daily functioning. |
| Impact of Event Scale (IES) | Horowitz et al., 1979 | - subjective distress following a specific traumatic or stressful life event. - assesses intrusion and avoidance. - goal is to measure stress responses rather than to provide a formal PTSD diagnosis. |
| Peritraumatic Distress Inventory (PDI-C) | Bui et al., 2011 | - emotional and physiological distress experienced during and immediately after a traumatic event. - assesses fear, helplessness, horror, and somatic reactions occurring at the time of trauma. - goal is early identification of children at increased risk for subsequent posttraumatic stress symptoms. |
| Peritraumatic Dissociative Experiences Questionnaire (PDEQ-C) | Bui et al., 2011 | - dissociative experiences occurring during or immediately after a traumatic event. - assess symptoms such as depersonalization, derealization, altered time perception, and emotional numbing. - purpose is to evaluate peritraumatic dissociation as a predictor of later PTSD development. |
| Child Report of Post-Traumatic Symptoms (CROPS) | Greenwald & Rubin, 1999 | - posttraumatic stress symptoms in children (DSM-IV). - assesses intrusion, avoidance, and hyperarousal. - goal is efficient identification of children who may require further trauma-focused assessment. |
